# Correlation between Leisure Activity Time and Life Satisfaction: Based on KOSTAT Time Use Survey Data

**DOI:** 10.1155/2018/5154819

**Published:** 2018-08-09

**Authors:** Yu-Jin Cha

**Affiliations:** Department of Occupational Therapy, Semyung University, Jecheon, Republic of Korea

## Abstract

**Rationale:**

This study aims to investigate the correlation between the available leisure activity time and life satisfaction of the healthy elderly and the factors affecting them.

**Method:**

For the analysis, data from the 2014 Time Use Survey (2014TUS) published by Statistics Korea (KOSTAT) was used. This study classified the detailed activities of 9228 subjects, based on the data in 2014TUS, and analyzed the differences in time use for occupation domains by age group.

**Results:**

It was found that a greater amount time used for outdoor leisure activities yielded a higher life satisfaction value. Differences were found in time use by occupation domains between younger and older groups. These showed higher life satisfaction for those with spouses, regular full-time jobs, higher education, and better health.

**Conclusion:**

Based on these results, in order to improve the quality of life (QoL) for older adults, it is necessary to develop various leisure programs that require dynamic physical activities and to prepare alternative policies at the national level to promote participation in leisure activities by older adults. This study will provide occupational therapists (OTs) with data they can use to help older adults who have difficulty in time usage through time management intervention to improve their life satisfaction and QoL.

## 1. Introduction

Unlike the time required for physiological needs or labor time, the quantity and nature of leisure time are not predetermined and may vary wildly depending on an individual's characteristics and tendencies. In particular, as the daily lives of older adults consist primarily of leisure time apart from the time required for physiological needs, their quality of life (QoL) can vary considerably depending on how they make use of their leisure time [1]. Besides having a lot of leisure time that they should manage somehow, the ways that older adults use their leisure time is fundamentally different from that of young people who use their leisure time for the purposes of reproducing and recovering from bodily and mental fatigue rather than for amusement [2].

For older adults, participation in leisure activities can help to provide solutions to the loneliness they have that results from loss of roles and can contribute to greater life satisfaction and happiness by providing chances for improving their self-esteem and self-realization. Life satisfaction values reflect the subjective level of individual satisfaction in achieving goals and expectations experienced in daily life [3]. Meaningful leisure activities are of critical importance because they are closely related to life satisfaction and quality of life for older adults in the contemporary era [4]. As the period spent in old age is getting longer, there is increasing concern regarding strategies for successful old age and improvement in the QoL for older adults [5]. Numerous studies regarding QoL for older adults have reported that participation in leisure activities contributes to maintaining and improving their physical health as well as their psychological and mental health and helps to maintain and increase their QoL by providing them with good opportunities for positive interaction with their families and others in society [6, 7].

Kelly argues that leisure activities are a more important factor than other experiences or activities, with regard to QoL [8]. Adams et al. asserted that a higher frequency of participation in leisure activities brings a higher QoL for older adults and allows them to live more happily [9]. That is, in order to maintain or improve older adults' QoL, it is most important to increase their opportunities to participate in leisure activities that can bring improvements in their physical, psychological, and social health [10].

Older adults should maintain a good balance in how they spend their time for various activities including self-care, leisure, rest, and social participation in order to improve their life satisfaction and health [11]. As the elderly population is increasing due to global demographic changes, it has become important to investigate older adults' time usage, that is, how they spend their days, in order to find ways to improve their QoL [12].

Occupational therapists (OTs) are experts in extending concern and encouraging participation by older adults in leisure activities, one of the fields of occupational therapy (OT) [4]. These global demographic changes will increase the number of older adults among OT clients, and consequently the contribution of OT to the lives of older adults will grow more and more important. In order for OTs to improve QoL for older adults, it is very important to investigate older adults' time usage, that is, how they spend their days [12]. If the status of older adults' time usage can be effectively analyzed, OTs will be able to suggest how older adults can participate actively in meaningful activities [1]. As daily life, which is closely related to temporal flow, proceeds in repeated cycles, one statistical source that can show the experiences and problems in daily living is time use survey data [13].

The investigation of time usage is necessary to evaluate the occupational balance that indicates how people subjectively choose mandatory and nonmandatory activities depending on personal values [14, 15]. Time management should be planned to provide an effective balance of private living, work, leisure, and rest to improve satisfaction and health [16]. The study of 24/7 living time usage enables us to investigate individual daily life and daily living within the social structure that surrounds individuals. In particular, such a temporally based approach is appropriate in dealing with older adults' daily living habits, which are difficult to study through official and economic approaches [13], as efficient time usage affects people's successful pursuit of meaningful activities and is an important criterion in deciding living modes, living aspects, and QoL [17]. In OT, studies regarding the time usage of individual persons such as those based on living time usage surveys are currently a primary concern and interest [18].

The *Occupational Therapy Practice Framework: Domain and Process (OTPF)* published by the American Occupational Therapy Association (AOTA) classifies various occupations in which a person or group participates into eight categories, namely, activities of daily living (ADL), instrumental activities of daily living (IADL), rest and sleep, education, work, play, leisure, and social participation, which are referred to as “occupation domains” [19]. For human beings, occupations are very critical elements, and each time a person participates in an occupation, an inevitable relation between occupation and time arises [20].

Looking into the causal relationship between the participation of the elderly population of South Korean in leisure activities and their life satisfaction mediated by leisure satisfaction that can be found in previous relevant literature, it is shown that the more older adults participated in leisure activities and were satisfied with them, the higher their life satisfaction [21]. Most of the studies analyzed the frequency of leisure activities by type or the subjective level of participation using a Likert scale. However, these research results have limitations in explaining precisely the relationship between increases in leisure activities and increases in the QoL and life satisfaction in the elderly of South Korea. Previous studies about life satisfaction in South Korea have focused on determining sociological and demographic variables affecting QoL, and there have been few studies analyzing the usage of leisure activity time as a variable. In addition, most of the previous studies have utilized limited sampling methods; therefore, their results were too limited to apply generally to the South Korean elderly population.

Even though South Korea entered the ranks of aged societies later than Europe or Japan, it is expected that by 2050, South Korea may become the most superaged society, as the portion of the population over 65 will increase dramatically [22]. Therefore, concern and interest regarding the comprehensive life satisfaction of older adults are likely to increase. This study investigated the correlation between the current status of leisure activity time use and life satisfaction of the healthy elderly in South Korea, factors affecting them, and the time usage of older adults as categorized according to the eight occupation domains suggested by OTPF-3 (OTPF 3rd Ed) and the aims at providing OTs with data so that they can help older adults who have difficulty in utilizing their time effectively through time management intervention aimed at improving their life satisfaction and QoL.

## 2. Method

### 2.1. Collecting Data

The 2014 Time Use Survey (2014TUS) used in this study was conducted by Statistics Korea (KOSTAT) in order to provide basic data for determining the 24-hour daily living modes and QoL of South Koreans, establishing policies related to labor, welfare, and transportation and conducting studies. A time use survey has been made every 5 years since 1999, and this study used the raw data collected in 2014 that measured South Koreans' lifestyles and QoL. For the analysis in this study, data regarding 9229 persons, the portion of the population aged 65 and over, was extracted from that of the 26,988 total subjects included in KOSTAT's 2014 Time Use Survey.

### 2.2. Overview of the Survey

The 2014 Time Use Survey (2014TUS) consists of a two-day diary record with respondents recording their primary activities and coactivities every 10 minutes. This analysis in this study included only the respondents' main activities. The respondents filled out the time diary with a record of activities based on classifications of activities (as of 2014: 9 major classifications, 42 medium classifications, and 138 minor classifications) covering their own activities as well as those of their companions, the location of the activities, and means of transportation involved, making an entry every 10 minutes for two days [23].

### 2.3. Subjects for the Analysis

The subjects for analysis were classified into a younger elderly group aged between 65 and 74 and an older elderly group aged 75 and over. Recently, as the life expectancy has increased, attempts have been made to subcategorize the age groups in studies on older adults. Neugarten et al. classified older adults into the younger elderly under 75 and the older elderly at 75 and older [24]. Compared to the younger elderly, the older elderly are more dependent on others due to their decreasing ability to conduct their daily life activities independently and have more difficulties in life due to the deaths of spouses and friends, economic hardship, deteriorating physical ability and health, and an increase in grave experiences in life [25]. The younger and the older elderly groups also differ in their respective health situations, which are affected by activities of daily living, family or social support, and health status. Therefore, when conducting studies and interventions related to them, different approaches should be taken for each group [26].

### 2.4. Classifications of Occupation Domains

Time use measuring instruments vary depending on research goals; therefore, activities were classified using a variety of methods. For this study, the subactivities included in the raw data of “2014 Time Use Survey” were categorized into a total of eight occupation domains (Appendix 1) [27]. Among these, leisure activities were classified into four subcategories, namely, indoor activities, outdoor activities, internet use, and other leisure activities (Table 1).

### 2.5. Analysis Method and Main Variables

For leisure time use in both elderly groups, frequency analysis was conducted, and in order to verify differences in the amount of time spent for leisure according to demographic characteristics, cross tabulation analysis (*χ* ^2^ test) and an *F* test using one-way ANOVA were conducted. In addition, for the differences in time usage by occupation domain in the South Korean elderly by age, Pearson's correlation coefficient test was conducted; for the correlation between leisure time spent and life satisfaction, analysis by averages was conducted; and for the correlation between leisure time spent, types of leisure activities, and life satisfaction, ordinary least squares (OLS) analysis was applied.

## 3. Results

Among the total of 9228 subjects, the younger elderly (at age 65–74) were 5436 (58.91%) and the older elderly (over 75 years) were 3792 (41.09%). For leisure activities, gender, educational attainment level, economic activity status, rural residence status, outdoor activities according to size of residential area, indoor activities, internet use, and other leisure activities showed significant differences (*p* < 0.05). Male older adults, those with higher educational attainment, those with nonworking status, those not living in rural areas, and those with urban/communal residence showed a significantly longer time usage in outdoor activities, indoor activities, internet use, and other leisure activities when their time spent on leisure activities was analyzed. For those with a spouse, the time for indoor activities and internet use was longer, while in the case of those with no spouse, the time for outdoor activities was longer (Table 1).

**Figure 1:**
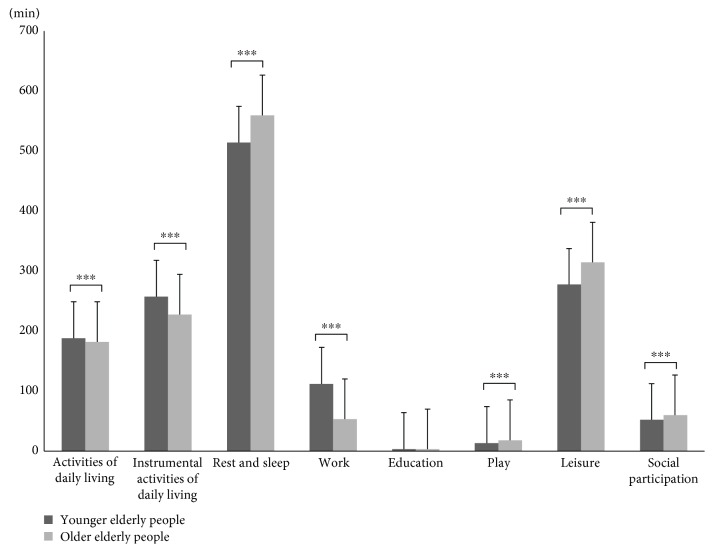
Differences of occupation domain time use between younger and older elderly groups (^∗∗∗^ *p* < 0.001).

In a review of differences in time usage by the South Korean elderly population by age in occupation domains including leisure and the seven domains other than education, that is, activities of daily living (ADL), instrumental activities of daily living (IADL), rest and sleep, work, play, leisure, and social participation, showed significant differences (*p* < 0.001). The younger elderly group showed higher time usage in the domains of ADL, IADL, and work, while the older elderly group showed higher time usage in the domains of rest and sleep, play, leisure, and social participation. Among the eight occupation domains, leisure ranked highest in both the younger and the older elderly groups, followed by indoor activities (Table 2, Figure 1).

The analysis of time use by the South Korean elderly showed that as their use of time for leisure activities increased, their life satisfaction was higher, and all controlled variables except for economic activity status exerted significant influences on life satisfaction. The subjects showed higher life satisfaction if they had spouses, regular full-time jobs, higher education, and better health. They had significantly longer time usage for outdoor activities, indoor activities, internet use, and other leisure activities if they were male, or had higher education, nonworking status, or residence in urban (communal) housing not in agricultural and rural areas (Table 3).

**Figure 2:**
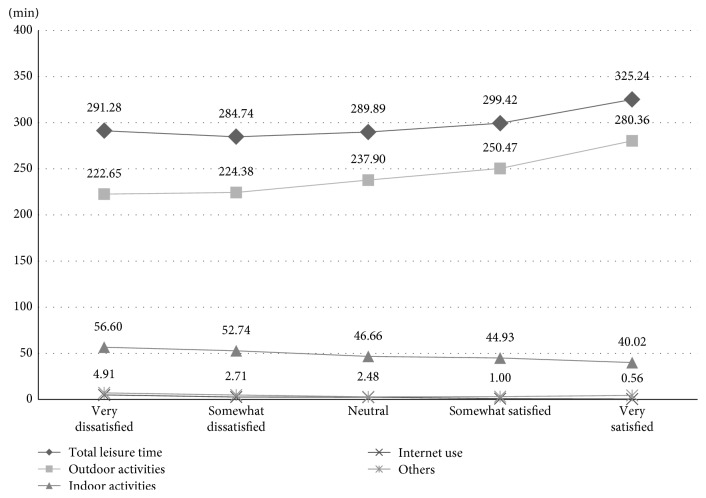
Life satisfaction in relation to length of leisure activity time and types of leisure activities.

The correlation between leisure time use and life satisfaction are shown in Table 4 and Figure 2. There is a general positive correlation between total time used for leisure and life satisfaction. For distribution of leisure time length according to a five-phase scale of life satisfaction level, in the case of very high life satisfaction, the average time spent for leisure activities was 325.24 minutes, while in the case of very low life satisfaction, the average daily time use for leisure activities was 291.28 minutes. Outdoor activities also showed a positive correlation, with longer leisure time used for outdoor activities corresponding to higher life satisfaction. However, in the case of indoor activities, an inverse correlation was shown, with longer time spent on indoor leisure activities corresponding to a lower life satisfaction.

## 4. Discussion

South Korean society has become a superaged society due to a rapid increase in the number of older adults, and concern for their comprehensive QoL is increasing. In this study, the usage of time by older adults over eight occupation domains suggested by OTPF-3 was studied, and correlations between leisure activity time use and life satisfaction were investigated to identify the satisfaction factors related to use of leisure time, and these factors were analyzed.

As a result of this study, it has been shown that there are differences in leisure activities related to gender, educational attainment, marital status, economic activities, residency in agricultural area all affecting outdoor activities according to size of residential area, indoor activities, internet use, and other leisure activities. It was shown that males and those with higher education, nonworking status, no residency in agricultural and rural areas, and urban (communal) residence recorded longer time usage in outdoor activities, indoor activities, internet use, and other leisure activities. In Noh's study, the female elderly in South Korea had the lowest perception of any demographic among the population regarding the use of leisure time, and their values and attitudes on leisure activities were negative [28]. Therefore, it is necessary to provide proper leisure education programs for the female elderly. On the other hand, Lee et al.'s study revealed that the female elderly spent more time than the male elderly in social leisure, voluntary works, and social activities [1]. The study noted that this was largely due to the fact that the female elderly spend comparatively more time participating in religious activities than the male elderly. Since religious activities are categorized as to IADL rather than leisure, the balance of the research outcome was affected by this categorization.

As a result of reviewing differences in time usage by occupation domains between the younger elderly and older elderly populations in South Korea, the seven domains other than education—ADL, IADL, rest and sleep, work, play, leisure, and social participation—showed significant differences. In addition, the younger elderly group showed higher time usage in ADL, IADL, and work, while the older elderly group showed higher time usage in rest and sleep, play, leisure, and social participation. Therefore, this study found out that methodologies for intervention in time management for older adults should differ according to age.

Among the eight occupation domains, leisure was the highest in both the younger and older elderly groups, and indoor activities were the highest among leisure activities. For these results, according to KOSTAT data as of 2008, the average retirement age in South Korea was 53, and life after retirement transitioned from a focus on work to one on leisure [29]. Regarding time used for rest and sleep by older adults, there was no change, with older adults using more time in rest and sleep than children and younger adults [15].

The South Korean elderly engage primarily in passive and static leisure activities, and this is borne out by the KOSTAT data [30]. The highest leisure activity for older adults was watching TV, followed by religion, cultural activities, and sports activities in that order. This result was also consistent with the previous studies [27, 31]. If older adults' leisure activities consist primarily of static ones such as watching TV, they are not activities with high meaning value. In order to improve older adults' QoL, it is necessary to develop various leisure programs that require dynamic physical activities, because this can relieve older adults of anxiety, help them regain their self-esteem, and help them maintain interpersonal relationships with those who participate together [32]. It is also necessary to implement alternative policies at national level in order to promote the participation of older adults' in leisure activities. Older adults in the western world including the USA, Canada, and the UK showed cultural differences from older adults in South Korea as they spend extensive time engaged in various sports activities such as golf, jogging, and swimming for their leisure [33, 34].

For the South Korean elderly, the longer their leisure time, the higher their life satisfaction. In addition, except for economic activity status, all control variables exert significant influence on life satisfaction. Study subjects showed higher life satisfaction if they had spouses, regular full-time jobs, higher education, and better health. They had significantly longer time usage for outdoor activities, indoor activities, internet use, and other leisure activities if they were male, or had higher education, nonworking status, and urban (communal) housing not in agricultural and rural areas. These results are consistent with previous studies and with ordinary expectations related to life satisfaction for older adults [35, 36].

This study did not show a significant influence on life satisfaction due to the status of economic activities, a finding which supports the previous studies that indicated cultural factors such as educational attainment or individual environment including area of residences could affect leisure activities more than economic factors including income and occupation. The reason for this is that education contributes to the ability to maintain a higher level of stimuli, cultures, or insights required for utilizing leisure time effectively [37]. In the capitalist social system, individual economic capability is required to maintain one's life, but it is appropriate to view it as an instrument rather than a requirement.

For the limitations of this study, first, differences in living time usage depending on individual characteristics or socioeconomic position were not considered since the statistical analysis was made by classifying the groups according to age. Second, the occupation domains cited by OTPF-3 were created in the US context; therefore, they may contain cultural differences from the actual domains of living time usage by the South Korean elderly. In future studies, it is necessary to compare the time usage of older adults who have health issues with that of healthy older adults in order to prepare a foundation for intervening with regard to time usage and also to further investigate the current status of space and facilities available for leisure activities.

It is expected that this study, which has analyzed KOSTAT's 2014TUS, will provide basic data for the academic community and staff persons charged with the care of the elderly to help improve older adults' life satisfaction and QoL by helping them to make optimal meaningful use of their time, as physical, social, psychological, and mental problems are bound to occur with increasing frequency due to the longer average life expectancy.

## 5. Conclusions and Implications for Occupational Therapy Practice

Although time is a resource identically distributed to everyone, depending on how it is utilized by a person, their individual performances and QoL can vary. In this study, we investigated the status of time usage of the healthy South Korean elderly through classification by eight occupation domains suggested by OTPF-3, along with the correlations between the status of leisure activity time use and life satisfaction, in order to identify characteristics of leisure activities and factors affecting them. As a result, it was shown that the South Korean elderly showed higher life satisfaction as the amount of time they spend in leisure activities is increased. In addition, while longer time for outdoor activities of leisure brought a higher life satisfaction, the opposite was true in the case of indoor activities. Differences were found in the distribution of time over occupation domains between the younger group and the older group of the South Korean elderly. This study has a significance in having analyzed a national time use data based on the 2014TUS by KOSTAT from an occupational perspective and suggests evidence for developing various leisure programs incorporating dynamic physical activities in order to improve older adults' QoL. Older adults should have the opportunity to increase their health and emotional support through various physical leisure activities such as hobbies, sports activities, and social gatherings, which means that programs requiring more dynamic physical activities should be developed. OTs are experts who can organize high-quality programs reflecting the physical abilities and needs of older adults and can help those in need of assistance to achieve a balanced life and participate as fully as possible in everyday tasks. OTs should develop leisure education programs for individuals that take into account the physical abilities of older adults, finding an optimal active attitude for older adults so that they can enjoy high-quality leisure life. This study will provide OTs with data they can use to assist older adults who have difficulty in time usage through time management intervention in order to improve their life satisfaction and QoL.

## Figures and Tables

**Table 1:** Differences of leisure activities in the Korean elderly according to demographic characteristics.

Items	Classifications	*N* (%)	Outdoor activities			Indoor activities			Internet use			Other		
			M	SD	$\frac{t}{F}$	M	SD	$\frac{t}{F}$	M	SD	$\frac{t}{F}$	M	SD	$\frac{t}{F}$
Age	Younger elderly (aged 65–74)	5436 (58.91)	50.53	70.22	23.89 *p* ≤ 0.001	220.69	140.88	221.16 *p* ≤ 0.001	2.99	17.58	30.20 *p* ≤ 0.001	3.42	17.22	1.11 *p* = 0.293
	Older elderly (aged 75 and over)	3792 (41.09)	43.57	62.90		265.96	148.02		1.21	11.32		3.86	23.09	
Gender	Male	3800 (41.18)	65.35	77.86	466.79 *p* ≤ 0.001	270.04	153.19	*297.50p* ≤ 0.001	4.90	22.86	*195.66p* ≤ 0.001	6.23	26.99	*114.28p* ≤ 0.001
	Female	5428 (58.82)	35.30	55.73		217.77	135.91		0.41	5.13		1.77	12.31	
Educational attainment	No educational attainment	1900 (20.59)	30.49	51.17	111.35 *p* ≤ 0.001	242.41	145.08	4.70 *p* = 0.001	0.06	1.47	121.30 *p* ≤ 0.001	1.88	10.94	41.27 *p* ≤ 0.001
	Elementary school	3498 (37.91)	40.77	62.34		237.25	142.64		0.25	5.08		2.07	11.57	
	Middle school	1500 (16.25)	51.24	68.72		230.01	144.82		1.75	13.76		3.11	15.13	
	High school	1520 (16.47)	66.49	77.79		240.21	152.21		5.06	22.67		5.99	24.49	
	Postsecondary education	810 (8.78)	75.90	79.69		256.30	146.54		11.75	33.46		10.72	44.73	
Marital status	Not married	3604 (28.11)	244.99	146.06	*9.07p* = 0.003	39.94	60.04	*78.55p* ≤ 0.001	0.52	6.91	*76.02p* ≤ 0.001	2.76	15.32	*10.67p* = 0.001
	Married	5624 (43.87)	235.64	145.14		52.63	71.27		3.37	18.78		4.14	22.25	
Reason for needing care	Care not needed	8654 (93.78)	47.97	67.92	1.68 *p* = 0.153	233.87	142.93	52.43 *p* ≤ 0.001	2.34	15.49	1.21 *p* = 0.305	3.69	20.29	0.83 *p* = 0.508
	Dementia	62 (0.67)	51.77	61.18		327.42	142.54		0.00	0.00		2.10	12.43	
	Stroke	40 (0.43)	59.50	75.38		392.00	176.64		1.50	9.49		2.25	14.23	
	Disability	102 (1.11)	42.75	70.19		327.84	173.16		0.78	7.92		0.78	5.57	
	Other reasons	370 (4.01)	40.22	52.27		310.54	155.43		1.08	15.33		2.84	11.35	
Economic activity status	Working	2.87 (31.19)	29.86	55.33	301.84 *p* ≤ 0.001	172.62	124.69	969.57 *p* ≤ 0.001	1.61	11.27	7.48 *p* = 0.006	2.68	14.64	9.01 *p* = 0.003
	Not working	6350 (68.81)	55.75	70.74		269.51	144.29		2.55	16.86		4.02	21.79	
Average monthly household income	Less than 1 million KRW	7080 (76.70)	45.60	65.53	*16.90p* ≤ 0.001	240.20	145.44	*0.94p* = 0.420	1.77	13.89	*12.36p* ≤ 0.001	3.54	19.73	*1.11p* = 0.346
	One million KRW–less than 2 million KRW	1420 (15.40)	51.76	70.82		233.60	150.40		4.12	19.26		4.22	21.70	
	2 million KRW–less than 3 million KRW	422 (4.60)	61.59	74.66		243.44	134.28		4.29	20.89		4.02	20.26	
	Over 3 million KRW	306 (3.30)	67.75	84.21		233.60	142.17		4.46	22.45		1.90	11.90	
Classifications of agricultural residence	Agricultural residence	1694 (18.36)	26.71	54.45	*205.19p* ≤ 0.001	198.71	131.90	*164.16p* ≤ 0.001	0.83	8.66	*17.92p* ≤ 0.001	1.68	11.18	*19.48p* ≤ 0.001
	Nonagricultural residence	7534 (81.64)	52.39	69.10		248.42	146.94		2.58	16.46		4.04	21.29	
Size of residential area^∗^	County/eup	2774 (30.06)	54.45	1.32	205.19 *p* ≤ 0.001	131.90	3.20	164.16 *p* ≤ 0.001	8.66	0.21	17.92 *p* ≤ 0.001	11.18	0.27	19.48 *p* ≤ 0.001
	Urban/dong	6454 (69.94)	69.10	0.80		146.94	1.69		16.46	0.19		21.29	0.25	
Period covered by research data	Weekdays	5464 (59.21)	47.06	67.05	1.10 *p* = 0.294	236.98	144.75	3.40 *p* = 0.065	2.18	14.74	0.34 *p* = 0.559	3.78	20.03	1.01 *p* = 0.314
	Weekend	3764 (40.79)	48.56	67.87		242.66	146.70		2.37	16.19		3.35	19.57	
Cohabitant status	Living alone	2330 (25.25)	232.16	143.83	7.49 *p* = 0.006	40.39	61.70	36.59 *p* ≤ 0.001	0.90	9.12	24.40 *p* ≤ 0.001	3.58	20.59	0.01 *p* = 0.937
	Having cohabitants	6898 (74.75)	241.70	146.08		50.13	69.04		2.72	16.92		3.61	19.58	

^∗^Based on KOSTAT criteria, “county/eup” is a designation use in agricultural and rural areas and “urban/dong” in urban areas.

**Table 2:** Differences in time use in occupation domains by the Korean elderly population by age (min).

Occupations	Over 65 years old		Age				*t*-test
			Younger elderly people (aged 65–74)		Older elderly people (aged 75 and over)		
	M	SD	M	SD	M	SD	
ADL	185.47	59.32	187.97	59.93	181.89	58.25	5.84^∗∗∗^
IADL	245.13	156.46	257.40	159.87	227.53	149.71	9.65^∗∗∗^
Rest and sleep	532.82	110.44	514.19	101.51	559.53	117.05	−17.67^∗∗∗^
Work	87.83	27.42	111.90	184.29	53.32	118.62	14.59^∗∗∗^
Education	3.36	20.29	3.39	20.59	3.32	19.85	1.16
Play	15.16	43.2	13.25	40.60	17.91	46.56	−4.77^∗∗∗^
Leisure							
Total leisure time	292.83	166.76	277.64	167.28	314.60	163.61	−8.68^∗∗∗^
Outdoor activities	47.67	67.39	50.53	70.22	43.57	62.90	
Indoor activities	239.29	145.57	220.69	140.88	265.96	148.02	
Internet use	2.26	15.35	2.99	17.58	1.21	11.32	
Others	3.60	19.84	3.42	17.22	3.86	23.09	
Social participation	55.41	65.04	52.28	62.71	59.89	67.99	−7.01^∗∗∗^
*N*	9228		5436		3792		

Note: ^∗∗∗^ *p* < 0.001.

**Table 3:** The influences of leisure time on life satisfaction.

Model	Nonstandardization coefficient		Standardization coefficient	*t*	Probability of significance	VIF
	*B*	Standard deviation	Beta			
(Constant)	3.393	.137		24.750	.000	
Leisure time	.000	.000	.027	2.320	.020	1.32
Age	−.007	.002	−.051	−4.578	.000	1.25
Gender	−.148	.023	−.080	−6.382	.000	1.58
Educational attainment	−.106	.008	−.166	−13.334	.000	1.56
Marital status	.074	.019	.044	3.934	.000	1.28
Reason for needing care	.177	.011	.165	16.123	.000	1.05
Economic activity status	−.002	.013	−.002	−.128	.898	1.69
Average monthly income	−.063	.006	−.126	−10.669	.000	1.40
Living in agricultural residence	.108	.031	.046	3.547	.000	1.69
Rural/urban residences	−.006	.004	−.017	−1.648	.099	1.03
Study conducted on weekdays	.068	.024	.034	2.865	.004	1.42
Elderly household code	.005	.004	.012	1.185	.236	1.00

*F* = 69.808 (*p* = 0.000).

**Table 4:** Level of life satisfaction in relation to the amount of leisure activity time and type of leisure activity.

	Very dissatisfied		Somewhat dissatisfied		Neutral		Somewhat satisfied		Very satisfied	
	M	SD	M	SD	M	SD	M	SD	M	SD
Total leisure time	291.28	169.16	284.74	163.91	289.89	164.02	299.42	169.62	325.24	183.29
Outdoor activities	222.65	140.25	224.38	140.59	237.90	141.87	250.47	150.64	280.36	168.86
Indoor activities	56.60	70.72	52.74	71.49	46.66	66.67	44.93	62.78	40.02	68.55
Internet use	4.91	23.64	2.71	15.60	2.48	16.60	1.00	9.60	0.56	6.10
Others	7.12	38.05	4.90	22.85	2.85	15.02	3.04	20.51	4.29	19.14

## Additional Points

*Concise Description*. For this study, subactivities found in the raw data of “2014 Time Use Survey” were categorized into a total of eight occupation domains (activities of daily living, instrumental activities of daily living, rest and sleep, work, education, play, leisure, and social participation). This was based on eight “occupation domains” suggested by OTPF-3.

## Supplementary Materials

Supplementary Materials Appendix 1: reclassification of occupation domain on detailed activities in Korean Time Use Survey (KTUS) 2014. For this study, subactivities in the raw data of “Time Use Survey 2014” were categorized into a total of eight occupation domains (activities of daily living, instrumental activities of daily living, rest and sleep, work, education, play, leisure, and social participation). It was based on eight “occupation domains” suggested by OTPF-3.
