# Supplementary material for: Correlation between Leisure Activity Time and Life Satisfaction: Based on KOSTAT Time Use Survey Data
Source: Occup Ther Int. 2018 Aug 9;2018:5154819. doi: 10.1155/2018/5154819 (PMC6109471; doi:10.1155/2018/5154819)
Supplement: Supplementary Materials — Appendix 1: reclassification of occupation domain on detailed activities in Korean Time Use Survey (KTUS) 2014. For this study, subactivities in the raw data of “Time Use Survey 2014” were categorized into a total of eight occupation domains (activities of daily living, instrumental activities of daily living, rest and sleep, work, education, play, leisure, and social participation). It was based on eight “occupation domains” suggested by OTPF-3. [file 5154819.f1.docx]

Appendix 1: Reclassification of occupation domain on detailed activities in 2014 Time Use Survey (2014TUS)

| Contents of activities | Code number | Occupation domain |
| --- | --- | --- |
| Sleeping | A120 | R |
| Insomnia | A140 | R |
| Sick leave | A340 | R |
| Idling | G970 | R |
| Eating | A220 | A |
| Snacking and drinking | A240 | A |
| Personal hygiene | A920 | A |
| Dressing, make-up | A940 | A |
| Other personal care activity | A990 | A |
| Class time | C120 | E |
| Breaks between classes | C140 | E |
| Self-study at school | C160 | E |
| School/University events | C180 | E |
| Other School/University activities | C190 | E |
| Taking courses at private institutions | C220 | E |
| Taking broadcasting/online courses | C240 | E |
| Self-study | C260 | E |
| Other education than school activities | C290 | E |
| Leisure and liberal arts learning | G940 | E |
| Self-therapy | A320 | I |
| Getting medical care services | A360 | I |
| Getting beauty services | A960 | I |
| Food preparation | D120 | I |
| Making snacks or desserts | D140 | I |
| Clearing table, washing dishes | D160 | I |
| Getting food-related services | D180 | I |
| Laundry | D220 | I |
| Fixing and producing homeware and shoes | D240 | I |
| Getting homeware and shoes related services | D280 | I |
| Cleaning | D320 | I |
| Tidying up home | D340 | I |
| Dumping trash | D360 | I |
| Household management | D420 | I |
| Household item management and production | D440 | I |
| Getting residence related service | D460 | I |
| Getting household item related services | D480 | I |
| Vehicle maintenance | D520 | I |
| Getting vehicle maintenance services | D540 | I |
| Pet care | D620 | I |
| Plant care | D640 | I |
| Getting pet and plant care services | D660 | I |
| Off-line shopping | D720 | I |
| On-line shopping | D740 | I |
| Offline purchase of service | D760 | I |
| Online purchase of service | D780 | I |
| Other shopping related behavior | D790 | I |
| Organizing housekeeping book | D920 | I |
| Using the services of financial institutions | D940 | I |
| Using the services of public office etc. | D960 | I |
| Other home maintenance | D990 | I |
| Physical Care (under 10 years old) | E120 | I |
| Teaching children (under 10 years old) | E140 | I |
| Reading books /Playing with children (under 10 years old) | E160 | I |
| Nursing children (under 10 only) | E180 | I |
| Other cares of children (under 10 only) | E190 | I |
| Physical Care (over 10 years old) | E220 | I |
| Studying (over 10 years old) | E240 | I |
| Nursing (over 10 years old) | E260 | I |
| Other care (over 10 years old) | E290 | I |
| Nursing (spouse) | E320 | I |
| Other care (spouse) | E390 | I |
| Nursing (parents and grandparents living together) | E420 | I |
| Other caring (parents and grandparents living together) | E490 | I |
| Nursing (other family members or roommate) | E520 | I |
| Other caring (other family members or roommate) | E590 | I |
| Nursing (parents and grandparents not living together) | E620 | I |
| Other caring (parents and grandparents not living together) | E690 | I |
| Nursing (other family members not living together) | E720 | I |
| Other caring (other family members not living together) | E790 | I |
| Helping housekeeping activity | F360 | I |
| Religious activities | G320 | I |
| Participating in religious gatherings | G340 | I |
| Other religious activities | G390 | I |
| Traveling due to personal care | H120 | I |
| Commuting | H220 | I |
| Traveling due to other works | H240 | I |
| Traveling due to learning | H320 | I |
| Traveling due to home maintenance | H420 | I |
| Traveling related to caring family members living together | H520 | I |
| Traveling related to caring family members not living together | H540 | I |
| Traveling related to volunteer/participation | H720 | I |
| Traveling related to fellowship and leisure activities | H820 | I |
| Others relevant to traveling | H920 | I |
| Reading books | G210 | L |
| Reading newspapers | G220 | L |
| Reading magazines | G230 | L |
| Watching TV | G240 | L |
| Watching Videos etc. | G250 | L |
| Listening to the radio | G260 | L |
| Listening to records/CDs/Tapes/MP3 | G270 | L |
| Internet surfing | G280 | L |
| Other leisure activities related to media | G290 | L |
| Smoking | G960 | L |
| Other leisure activities | G990 | L |
| Personal hobbies | G930 | L |
| Movie theatre / Video room | G410 | L |
| Concert/theatre | G420 | L |
| Visiting museum/exhibition halls | G430 | L |
| Watching sports games | G440 | L |
| Tourism / Driving tour | G450 | L |
| Other cultural and tourism activities | G490 | L |
| Walking | G510 | L |
| Running/ Jogging | G520 | L |
| Climbing | G530 | L |
| Bicycle / In-line skate | G540 | L |
| Personal exercise | G550 | L |
| Ball game | G560 | L |
| Fishing / Hunting | G570 | L |
| Other sports / Leports | G590 | L |
| Mass game / plays | G910 | P |
| Computer / mobile games | G920 | P |
| Entertainment | G950 | P |
| Mandatory participation | F120 | S |
| Other participation | F190 | S |
| Caring for your acquaintances | F320 | S |
| Helping income-generating activities | F340 | S |
| Other helping | F390 | S |
| Face-to-face encounter | G120 | S |
| Interactions via video and voice exchange | G140 | S |
| Interactions via text and mail exchange | G160 | S |
| Other fellowship activities | G190 | S |
| Ceremonies such as wedding, funeral etc. | G620 | S |
| Major job | B110 | W |
| Side jobs | B120 | W |
| Unpaid agriculture, forestry and fishery work on family farm | B130 | W |
| Unpaid work on family farm besides agriculture, forestry and fishery | B140 | W |
| Agriculture, forestry and fishery work for self-consumption | B150 | W |
| Break at workplace | B170 | W |
| Work related training | B180 | W |
| Other job-related activities | B190 | W |
| Job seeking | B220 | W |
| Participation related to children's education | F140 | W |
| Volunteering for national and local events | F220 | W |
| Activities related to the neglected class | F240 | W |
| Activities related to people in disaster | F260 | W |
| Other volunteering work | F290 | W |

Note: A, activities of daily living; I, instrumental activities of daily living; R, rest and sleep; W, work; E, education; P, play; L, leisure; S, social participation
